# The Association between COVID-19 and Changes in Opioid Prescribing Patterns and Opioid-Related Overdoses: A Retrospective Cohort Study

**DOI:** 10.1080/24740527.2023.2176297

**Published:** 2023-04-06

**Authors:** Alexandra Robins, Alan Dimitriev, Cameron MacKay, Hayden Wang, Abigail Kearney, Daniel P. Borschneck, Amber Simpson

**Affiliations:** a Department of Molecular and Biomedical Sciences, Queen’s University, Kingston, Ontario, Canada; b School of Computing, Queen’s University, Kingston, Ontario, Canada; c Department of Surgery, Queen’s University, Kingston, Ontario, Canada; d Division of Orthopaedic Surgery, Kingston Health Sciences Centre, Queen’s University, Kingston, Ontario, Canada

**Keywords:** Opioids, prescriptions, COVID-19, prescribing specialist

## Abstract

**Background:**

Recent data suggest that restrictions related to COVID-19 resulted in changes in the prescribing patterns of opioids.

**Aims:**

We sought to analyze Ontario health data for changes in frequencies among new and continuing users for the following opioid prescription characteristics: the type of opioid, the average daily dose, and the prescriber’s specialty.

**Methods:**

Utilizing data on the Ontario Health Data Platform, we defined two 149-day windows as “before” and “after” based on the initial COVID-19 provincial lockdown. A total of 882,268 individuals met our inclusion criteria and were classified as either “new” or “continuing” users. Chi-square tests and Fisher’s exact tests were applied for each level of our primary outcomes to determine whether there were significant changes in prescription proportions before and after the lockdown.

**Results:**

A decline of 28% was observed for the number of new users after the lockdown. Statistically significant changes were observed for new users across almost all opioid prescription characteristics between the before and after windows. The proportion of new users who received at least one dispensing event from a pharmacist increased by 26.32%, whereas continuing users increased by 378.61%. There were no statistically significant shifts in opioid prescriptions among individuals with a reported toxicity event during the study period.

**Conclusions:**

In terms of opioid prescribing patterns, new users experienced greater change following the onset of the pandemic lockdown than continuing users. Our findings potentially showcase the unintended impacts that COVID-19-related restrictions had on non-COVID-19-related health services, which can inform future policy decisions.

## RÉSUMÉ

**Contexte:** Des données récentes indiquent que les restrictions liées à la COVID-19 ont entrainé des changements dans la prescription des opioïdes.

**Objectifs:** Nous avons cherché à analyser les données sur la santé de l’Ontario pour déceler les changements de fréquence chez les nouveaux utilisateurs et les utilisateurs prévalents pour les caractéristiques de prescription d’opioïdes suivantes : le type d’opioïde, la dose quotidienne moyenne, et la spécialité du prescripteur.

**Méthodes:** En utilisant les données de la plateforme de données sur la santé de l’Ontario, nous avons défini deux fenêtres de 149 jours comme suit : « avant » et « après » le confinement provincial initial de la COVID-19. Un total de 882 268 personnes ont répondu à nos critères d’inclusion et ont été classées comme « nouveaux utilisateurs » ou « utilisateurs prévalents ». Des tests de chi-carré et des tests exacts de Fisher ont été appliqués pour chaque niveau de nos résultats primaires afin de déterminer s’il y avait eu des changements importants dans les proportions prescrites avant et après le confinement.

**Résultats:** Une baisse de 28 % a été observée pour le nombre de nouveaux utilisateurs après le confinement.

Des changements statistiquement significatifs ont été observés pour les nouveaux utilisateurs pour presque toutes les caractéristiques de prescriptions d’opioïdes entre les fenêtres avant et après. La proportion de nouveaux utilisateurs ayant eu au moins une prescription remplie par un pharmacien a augmenté de 26,32 %, tandis que le nombre d’utilisateurs prévalents a augmenté de 378,61 %. Il n’y a pas eu de changements statistiquement significatifs dans les prescriptions d’opioïdes parmi les personnes ayant déclaré un évènement de ⁠toxicité au cours de la période d’étude.

**Conclusions:** En matière de modèles de prescription d’opioïdes, les nouveaux utilisateurs ont connu un changement plus important après le début du confinement de la pandémie que les utilisateurs prévalents. Nos résultats démontrent possiblement les répercussions inattendues des restrictions liées à la COVID-19 sur les services de santé non liés à la COVID-19, ce qui pourrait éclairer les décisions politiques futures.

## Introduction

Following the onset of the COVID-19 pandemic, significant increases in the number of opioid-related deaths were reported in Ontario.^1^ During this time, a reduction in opioid prescribing for opioid-naïve patients was observed, whereas existing patients seemed to maintain the same level of access to opioids.^2^ Despite these findings, there remains a distinct lack of published literature that focuses on the pandemic’s impact on opioid prescribing. Important changes to prescribing regulations occurred during the initial lockdown, including an exemption to Subsection 56(1) of the Controlled Drugs and Substances Act issued in March 2020, which allowed pharmacists in Ontario to prescribe, sell, or provide controlled substances in limited circumstances or transfer prescriptions for controlled substances.^3^ The impact of these COVID-19 regulations remains unknown, and this gap in current knowledge signifies the need to investigate the patterns of opioid prescribing to inform appropriate public health policy decisions.

How regulation changes impacted patterns in distinct prescriber specialties, opioid types, amount of opioids prescribed, or opioid-related overdose rates remains unknown. Investigating these prescription characteristics is important given that the risk of opioid overdose has been reported to depend on these factors.^4,5^ This study aims to investigate how the distribution of these prescription characteristics may have changed following the onset of COVID-19-related lockdowns. A second aim is to investigate significant changes in the prescribing patterns of individuals who have experienced an opioid toxicity event. To do this, we use the Ontario Health Data Platform (OHDP), which was developed at the onset of the pandemic to provide researchers with streamlined access to large health care–related data sets to study trends in health services relating to the pandemic.^6^

## Methods

### Data Summary

All data sets used in this study were curated and provided by ICES and were accessed through the OHDP, an integrated and secure data platform re-created by the government of Ontario in response to the COVID-19 pandemic.^6^ ICES is a prescribed entity under Ontario’s Personal Health Information Protection Act (PHIPA). Section 45 of PHIPA authorizes ICES to collect personal health information, without consent, for the purpose of analysis or compiling statistical information with respect to the management of, evaluation or monitoring of, the allocation of resources to, or planning for all or part of the health system. Projects that use data collected by ICES under Section 45 of PHIPA, and use no other data, are exempt from research ethics board review. The use of the data in this project is authorized under Section 45 and approved by ICES’ Privacy and Legal Office. Information regarding prescription drug claims was retrieved from the Narcotics Monitoring System (NMS). Physician subspecialties (e.g., general practitioner) were identified using ICES Physician Database; for a full list of the subspecialties categorized as “other physicians” please see Table 1 in the supplementary materials. Emergency department visits for opioid toxicity events were identified using the Canadian Institute for Health Information (CIHI) National Ambulatory Care Reporting System (NACRS). Patient demographic information was collected from the Registered Persons Database. Individuals who changed classifications during the study period or had missing values for income quintile and residence were classified as “other.” CIHI’s Discharge Abstract Database (DAD), the Ontario Mental Health Reporting System, and NACRS were used to amalgamate mental health diagnoses covering a time frame between October 20, 2016, and October 20, 2019. Data retrieved from DAD were used in conjunction with data from the Ontario Health Insurance Plan to identify individuals who used palliative care services in a 365-day lookback period from their index prescription. Assessments recorded in the Continuing Care Reporting System were used to identify individuals who had utilized long-term care services up to 120 days prior to their prescribing date. Research ethics board approval was not required because all data sets used in the study (NMS, NACRS, DAD, etc.) were collected by ICES. No other data sets were used other than those collected by ICES. All data processing and analysis in this study were performed through the OHDP using Python v3.9.2 via Jupyter Notebook v6.2.0. utilizing NumPy 1.20.1, Pandas 1.2.3, and SciPy 1.6.0. These data sets were linked using unique encoded identifiers.

**Table 1.** Baseline characteristics of people receiving opioid prescriptions in the before and after time frames.

		New users				Continuing users			
		Before, *N* (%)		After, *N* (%)	% Change	Before, *N* (%)		After, *N* (%)	% Change
Total number of individuals									
Total individuals		354,991 (58.06)		256,471 (41.94)	−27.75	262,758 (97.03)		246,386 (90.98)	−6.23
Demographic information									
Sex									
Females		192,954 (54.35)		141,525 (55.18)	1.52	146,050 (55.58)		137,105 (55.65)	0.11
Males		162,037 (45.65)		114,946 (44.82)	−1.81	116,708 (44.42)		109,281 (44.35)	−0.14
Birth year									
1954+		103,366 (29.12)		79,906 (31.16)	7	113,355 (43.14)		104,200 (42.29)	−1.97
1955–1974		121,217 (34.15)		83,471 (32.55)	−4.69	122,449 (46.6)		116,777 (47.4)	1.7
1975–1994		93,404 (26.31)		66,269 (25.84)	−1.8	26,330 (10.02)		24,827 (10.08)	0.56
1995–2004		37,004 (10.42)		26,825 (10.46)	0.34	624 (0.24)		582 (0.24)	−0.53
Income quintile									
1 (lowest)		69,704 (19.64)		52,481 (20.46)	4.21	69,922 (26.61)		69,209 (28.09)	5.56
2		70,172 (19.77)		51,706 (20.16)	1.99	56,149 (21.37)		56,210 (22.81)	6.76
3		71,199 (20.06)		51,173 (19.95)	−0.52	46,707 (17.78)		46,760 (18.98)	6.77
4		70,833 (19.95)		50,090 (19.53)	−2.12	39,988 (15.22)		39,937 (16.21)	6.51
5 (highest)		71,196 (20.06)		50,152 (19.55)	−2.5	33,753 (12.85)		33,561 (13.62)	6.04
Other income		1887 (0.53)		869 (0.34)	−36.26	16,239 (6.18)		709 (0.29)	−95.34
Residence									
Rural		39,451 (11.11)		29,258 (11.41)	2.65	39,037 (14.86)		38,275 (15.53)	4.56
Urban		314,474 (88.59)		226,441 (88.29)	−0.33	220,622 (83.96)		207,534 (84.23)	0.32
Other		1066 (0.3)		772 (0.3)	0.24	3099 (1.18)		577 (0.23)	−80.14
Comorbidities									
Mental health diagnoses									
Any mental health disorder		14,849 (4.18)		11,997 (4.68)	11.83	15,568 (5.92)		14,289 (5.8)	−2.12
Substance use–related disorder		4764 (1.34)		3823 (1.49)	11.07	5601 (2.13)		5090 (2.07)	−3.08
Schizophrenia		1105 (0.31)		905 (0.35)	13.36	1226 (0.47)		1112 (0.45)	−3.27
Mood disorder		4244 (1.2)		3449 (1.34)	12.49	4061 (1.55)		3719 (1.51)	−2.34
Anxiety disorder		4453 (1.25)		3607 (1.41)	12.12	4029 (1.53)		3724 (1.51)	−1.43
Trauma/stressor-related disorder		3259 (0.92)		2750 (1.07)	16.8	3085 (1.17)		2830 (1.15)	−2.17
Obsessive-compulsive disorder and related disorders		53 (0.01)		47 (0.02)	22.74	21 (0.01)		18 (0.01)	−8.59
Personality disorder		776 (0.22)		649 (0.25)	15.76	757 (0.29)		703 (0.29)	−0.96
Deliberate self-harm		2057 (0.58)		1718 (0.67)	15.6	2507 (0.95)		2279 (0.92)	−3.05
Use of services									
Care services									
Palliative care		9443 (2.66)		9761 (3.81)	43.07	9755 (3.71)		8687 (3.53)	−5.03
Long-term care		7661 (2.16)		7357 (2.87)	32.92	11,295 (4.3)		9775 (3.97)	−7.71

The before window corresponds to dispensing dates between October 20, 2019, and March 16, 2020. The after window corresponds to dispensing dates between March 17, 2020, and August 12, 2020. Mental health diagnoses correspond to discharge dates ranging from October 20, 2016, to October 20, 2019.

### Exposure

The initial COVID-19-related “lockdown” in the province of Ontario in 2020 was used as the exposure for this study. On March 17, 2020, the province declared a state of emergency and ordered most businesses to close.^7^ On August 12, 2020, all regions in Ontario joined stage 3 of “reopening.”^7^ Thus, COVID-19-related lockdown measures were in place from March 17, 2020, to August 12, 2020, defining a 149-day window after the initial lockdown that represents our “after” window. For comparison, a period of 149 days prior to the lockdown date (October 20, 2019, to March 16, 2020) was defined as the “before” window.

### Identification of the Cohort

Our study analyzes individuals receiving opioid therapy in Ontario. The following exclusions were applied: individuals residing outside of Ontario on the date of their index prescription, individuals residing outside of Ontario on the date of their most recent dispensing event preceding lockdown (if applicable), individuals with missing or invalid data on their age and/or sex, individuals with an invalid Ontario Health Insurance Plan number, and individuals who were younger than 15 years or older than 105 years at the beginning of the study period.

We defined two types of prescription opioid users in our study: new and continuing users. New users were defined as individuals who did not have any opioid prescriptions in the 365 days prior to their index prescription date. Continuing users were defined as individuals who had more than 90 days of ongoing prescription opioid therapy with a date of service before March 17, 2020, and who were not recorded as having a discontinuation of opioid therapy by ICES. Discontinuation is based on the absence of opioid prescriptions in the 180 days beyond the amount supplied in the previous prescription where the “previous prescription” is their most recent prescription relative to 180 days before the lockdown (September 19, 2019). Individuals who had less than 180 days between the amount supplied in their last prescription before the lockdown and the lockdown date itself were classified as continuing users. To ensure exclusivity between the defined user groups, we excluded new users who, over the time frame of the study, could also be classified as continuing users from the analysis. We investigated opioid prescriptions for codeine (both codeine and codeine combination formulations), hydromorphone, morphine, oxycodone (both oxycodone and oxycodone combination formulations), and tramadol; prescriptions for fentanyl and meperidine were excluded from the study due to the sparsity of the data and the associated risk of re-identification. It should be noted that ICES defined the opioid classes previously listed based on a resource referred to as The Ontario Drug Policy Research Network for the NMS. These identified drug classes capture several different formulations and brands. For example, the generalized drug class “morphine” is representative of morphine sulfate, morphine, and morphine HCl formulations, thus encapsulating commonly prescribed brands, such as (but not limited to) Kadian, Statex, and MS Contin. The drug classes of buprenorphine and methadone were excluded given that they are used for opioid dependency and this study focuses on prescription for treating pain. Furthermore, we identified individuals within our study who had an opioid toxicity event using the *International Statistical Classification of Diseases and Related Health Problems* 10th Revision codes T40.0 to T40.4 and T40.6 in NACRS. Only toxicity events that occurred during our study time frame were included in the analysis.

### Outcomes

Our study analyzed the changes in frequencies of three prescription characteristics: the type of opioid prescribed, the average daily dose in morphine milligram equivalents (MME), and the prescriber’s specialty. We compared the frequencies of these prescription characteristics before and after the initial lockdown for both user groups to identify any shifts in prescribing patterns. We also investigated these characteristics separately for individuals who had experienced an opioid toxicity event during the study period.

### Statistical Analyses

For each of the characteristics presented, percentage change was calculated as the proportional change between the percentage of the population attributed in the before and after windows. The chi2_contingency function from SciPy.stats was used to perform the chi-square test of independence. For a given 2 × 2 contingency table, if any of the cells had expected values less than five, the fishers_exact function from SciPy.stats was used to perform a Fisher’s exact test instead of a chi-square test. If any of the observed or expected values were less than one, neither test was applied and a *P* value of “N/A” was recorded. All tests used a type 1 error rate threshold of 0.05 for statistical significance. We applied these tests for each level of our primary outcomes to determine whether the proportions were significantly different before and after the lockdowns.

## Results

**Figure 1.**
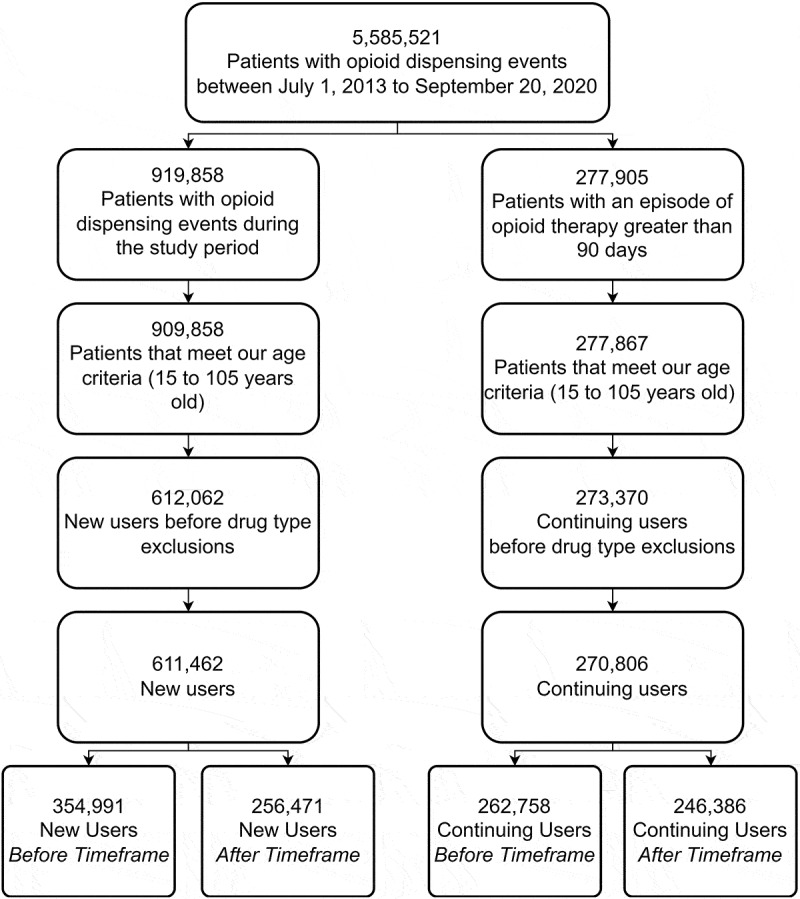
Cohort selection flowchart.

The baseline characteristics of the 882,268 individuals who met our inclusion criteria (Figure 1) for either user group are shown in Table 1. The total number of new users decreased from 354,991 in the “before” window to 256,471 in the “after” window, representing a decrease of approximately 28%. Continuing users also saw a decline in the total number of users; however, the percentage change for this user group was only −6.23%. There was no distinct change over the duration of the study for the distribution of sex. Mental health diagnoses were only observed in 4% of both user groups in either time frame. Long-term care and palliative care patients were also relatively uncommon.

Changes between the before and after windows for both user groups with regards to prescription characteristics are shown in Table 2. Statistically significant changes are observed across all opioid types for new users. The largest changes corresponded to the proportion of individuals prescribed oxycodone, which decreased by 8.44% (*P* < 0.001), and the proportion of individuals prescribed morphine and hydromorphone, which increased by 11.53% (*P* < 0.001) and 9.53% (*P* < 0.001), respectively. Conversely, only three types of opioids—codeine, hydromorphone, and tramadol—had significant changes among continuing users. Overall, in terms of the types of opioids being prescribed, the magnitude of percentage change was higher on average in new users than in continuing users. Regarding a patient’s average daily dose, decreases of approximately 15.71% (*P* < 0.001) and 26.48% (*P* < 0.001) were observed for the proportion of new users belonging to the >50–90 MME and >90–200 MME categories, respectively. There was an increase of approximately 68.31% (*P* < 0.001) in the proportion of new users who were missing dosage information. There were no statistically significant changes in the distribution of average daily dose categories for continuing users. Among new users, there were notable increases in the proportions of individuals with prescriptions from general practitioners (18.29%, *P* < 0.001), nurses (18.99%, *P* < 0.001), and pharmacists (26.32%, *P* < 0.001). Conversely, there were decreases in the proportions of individuals with prescriptions from dentists (−6.83%, *P* < 0.001) and from physicians classified as “other” (−14.41%, *P* < 0.001) among new users. Among continuing users, decreases in the proportion of individuals with at least one prescription occurred for physicians and across each of the physician subspecialties, with most of these changes being statistically significant. We observed decreases of approximately 24.15% (*P* < 0.001) in the proportion of continuing users who received at least one prescription from a dentist and approximately 3.74% for prescriptions from nurses (*P* < 0.001). The proportion of continuing users who received at least one prescription from a pharmacist increased from 0.24% to 1.13%, which represents a 378.61% change (*P* < 0.001). There were no statistically significant shifts in opioid prescriptions among individuals with a reported toxicity event during the study period (Table 3).

**Table 2.** Prescription opioid characteristics in the before and after time frames.

	New users				Continuing users			
	Before, *N* (%)	After, *N* (%)	% Change	*P* value	Before, *N* (%)	After, *N* (%)	% Change	*P* value
Opioid characteristics								
Type of opioid								
Codeine	180,054 (50.72)	126,813 (49.45)	−2.51	<0.001	88,337 (33.62)	80,314 (32.6)	−3.04	<0.001
Hydromorphone	77,642 (21.87)	61,438 (23.96)	9.53	<0.001	59,593 (22.68)	53,444 (21.69)	−4.36	<0.001
Morphine	24,919 (7.02)	20,079 (7.83)	11.53	<0.001	21,930 (8.35)	20,172 (8.19)	−1.9	0.04
Oxycodone	55,059 (15.51)	36,421 (14.2)	−8.44	<0.001	111,680 (42.5)	105,733 (42.91)	0.97	0.003
Tramadol	40,892 (11.52)	28,148 (10.98)	−4.72	<0.001	25,867 (9.84)	23,079 (9.37)	−4.85	<0.001
Average daily dose (MME)								
0–20	109,374 (30.81)	85,221 (33.23)	7.85	<0.001	97,960 (37.28)	91,905 (37.3)	0.05	0.886
>20–50	187,152 (52.72)	133,487 (52.05)	−1.28	<0.001	98,249 (37.39)	91,608 (37.18)	−0.56	0.121
>50–90	44,394 (12.51)	27,035 (10.54)	−15.71	<0.001	37,579 (14.3)	35,333 (14.34)	0.27	0.696
>90–200	9054 (2.55)	4809 (1.88)	−26.48	<0.001	19,177 (7.3)	18,209 (7.39)	1.26	0.21
>200	424 (0.12)	334 (0.13)	9.03	0.252	9203 (3.5)	8730 (3.54)	1.16	0.435
Missing	4593 (1.29)	5585 (2.18)	68.31	<0.001	590 (0.22)	601 (0.24)	8.63	0.161
Prescriber information								
Prescriber specialty								
Physician	277,341 (78.13)	202,755 (79.06)	1.19	<0.001	257,933 (98.16)	241,576 (98.05)	−0.12	0.002
General practitioner	122,244 (34.44)	104,473 (40.73)	18.29	<0.001	239,152 (91.02)	223,911 (90.88)	−0.15	0.087
Emergency physician	30,247 (8.52)	23,743 (9.26)	8.65	<0.001	9855 (3.75)	8067 (3.27)	−12.7	<0.001
Critical care physician	217 (0.06)	172 (0.07)	9.71	0.391	91 (0.03)	45 (0.02)	−47.26	<0.001
Other physician	140,975 (39.71)	87,170 (33.99)	−14.41	<0.001	35,020 (13.33)	28,255 (11.47)	−13.96	<0.001
Dentist	72,688 (20.48)	48,928 (19.08)	−6.83	<0.001	3647 (1.39)	2594 (1.05)	−24.15	<0.001
Pharmacist	515 (0.15)	470 (0.18)	26.32	<0.001	619 (0.24)	2778 (1.13)	378.61	<0.001
Nurse	8981 (2.53)	7721 (3.01)	18.99	<0.001	10,751 (4.09)	9704 (3.94)	−3.74	0.006

The before window corresponds to dispensing dates between October 20, 2019, and March 16, 2020. The after window corresponds to dispensing dates between March 17, 2020, and August 12, 2020. Number of people for type of opioid and prescriber specialty reflects that the person had at least one dispensing event with that given classification.

**Table 3.** Prescription opioid characteristics in the before and after time frames among individuals who had at least one emergency department visit for an opioid toxicity event.

	New users				Continuing users			
	Before, *N* (%)	After, *N* (%)	% Change	*P* value	Before, *N* (%)	After, *N* (%)	% Change	*P* value
Total individuals								
Emergency department visit for an opioid toxicity event	184 (0.05)	219 (0.09)	64.74		411 (0.16)	413 (0.17)	7.16	
Type of opioid								
Codeine	59 (32.07)	61 (27.85)	−13.13	0.417	67 (16.3)	73 (17.68)	8.43	0.666
Hydromorphone	75 (40.76)	93 (42.47)	4.18	0.807	198 (48.18)	174 (42.13)	−12.55	0.094
Morphine	38 (20.65)	43 (19.63)	−4.93	0.897	79 (19.22)	80 (19.37)	0.78	0.973
Oxycodone	29 (15.76)	35 (15.98)	1.4	0.939	198 (48.18)	184 (44.55)	−7.52	0.331
Tramadol	9 (4.89)	13 (5.94)	21.36	0.811	23 (5.6)	19 (4.6)	−17.79	0.623
Average daily dose (MME)								
0–20	43 (23.37)	52 (23.74)	1.6	0.976	66 (16.06)	63 (15.25)	−5.01	0.825
>20–50	83 (45.11)	97 (44.29)	−1.81	0.949	128 (31.14)	144 (34.87)	11.96	0.288
>50–90	29 (15.76)	26 (11.87)	−24.67	0.324	81 (19.71)	84 (20.34)	3.2	0.889
>90–200	12 (6.52)	19 (8.68)	33.03	0.535	85 (20.68)	71 (17.19)	−16.88	0.234
>200	17 (9.24)	25 (11.42)	23.56	0.583	50 (12.17)	50 (12.11)	−0.48	0.936
Prescriber specialty								
Physician	160 (86.96)	185 (84.47)	−2.85	0.572	406 (98.78)	407 (98.55)	−0.24	0.994
General practitioner	84 (45.65)	90 (41.1)	−9.98	0.413	370 (90.02)	365 (88.38)	−1.83	0.516
Emergency physician	35 (19.02)	37 (16.89)	−11.18	0.671	51 (12.41)	34 (8.23)	−33.66	0.063
Critical care physician	≤5	≤5	—	—	≤5	≤5	—	—
Other physician	67 (36.41)	90 (41.1)	12.86	0.391	110 (26.76)	95 (23)	−14.05	0.243
Dentist	20 (10.87)	24 (10.96)	0.82	0.895	11 (2.68)	8 (1.94)	−27.62	0.635
Pharmacist	≤5	≤5	—	—	≤5	≤5	—	—
Nurse	12 (6.52)	15 (6.85)	5.02	0.945	30 (7.3)	23 (5.57)	−23.7	0.384

The before window corresponds to dispensing dates between October 20, 2019, and March 16, 2020. The after window corresponds to dispensing dates between March 17, 2020, and August 12, 2020. Number of people for type of opioid and prescriber specialty reflects that the person had at least one dispensing event with that given classification.

## Discussion

Consistent with a previous study, our research has shown that there was a significant reduction in the number of opioid-naïve individuals receiving opioid prescriptions after the onset of the pandemic.^2^ However, a less dramatic decrease was seen for the number of individuals receiving opioid prescriptions among continuing users. Together, this indicates that following the initial COVID-19 lockdown the number of new opioid users was subject to greater change than the number of existing users already receiving opioid therapy.

Shifting prescribing trends were not limited to the number of patients receiving prescription opioids, because there were also shifts in the characteristics of the prescriptions themselves. For example, the proportion of individuals who had at least one prescription with missing dosage information increased by 68.31% for new users and 8.63% for continuing users. In the curated data set provided by ICES, the only prescription events that were missing MME dosage information were those identified with formulations of either injectables, suppositories, or some transdermal patches. Thus, the increases observed for the number of patients with missing morphine equivalent information could indicate that after the lockdown measures were put in place, patients were more frequently prescribed drug formulations that cannot be converted to MME or that patients who received such formulations were not as limited in their access to treatment. Across each of the average daily dose classifications, the percentage change for new users was larger in magnitude relative to the corresponding classification for continuing users. Comparatively, a recent study found that the weekly total MME for opioid-naïve users was 27% lower for March 18 to May 20, 2020, relative to the projected levels based on 2019 data. This is likely attributable to the 34% decrease in the weekly number of opioid-naïve patients during that same period and is similar to the 27.75% decrease in the number of new users in our study.^2^ Also consistent with our observations for continuing users, Currie et al. found that the levels for the weekly number of patients and total MME for existing patients were relatively similar for 2019 and 2020, suggesting that existing patients experienced limited disruptions in their care.^2^ These findings suggest that perhaps COVID-19-related restrictions not only potentially affected the number of new users more than the number of continuing users, but also potentially impacted the drug formulation and average daily doses that patients were prescribed.

Though greater differences among new users were observed, there was an interesting change among continuing users. The proportion of continuing users who received at least one prescription from a pharmacist increased from 0.24% to 1.13%; this corresponds to a change of 378.61%. The percentage increase among new users who received at least one prescription from a pharmacist was only 26.32%. The increase in the number of patients who received an opioid prescription from a pharmacist may be partially explained by a change in regulations in March 2020, when there was an exemption to Subsection 56(1) of the Controlled Drugs and Substances Act issued due to the COVID-19 pandemic. This allowed pharmacists in Ontario to prescribe, sell, or provide controlled substances in limited circumstances or transfer prescriptions for controlled substances (specifically allowing pharmacists to extend and renew opioid prescriptions, as well as transfer prescriptions to other pharmacists).^3^

The final notable finding of our study was that there were no statistically significant changes in the prescribing patterns among individuals in our study population who experienced an opioid toxicity event. This indicates that among individuals who experienced an opioid toxicity event, there were no changes in the prescription opioid characteristics that we analyzed before and after the COVID-19-related restrictions.

### Limitations

Strengths in our research are centered on the depth of our data set and its relevance to both the opioid crisis and the COVID-19 pandemic. Our study is not without limitations, the first of which is that the timing of lifting COVID-19 restrictions and reopening varied by municipality in Ontario. Because our data are province-wide, regional-level differences in the lockdown measures were not accounted for. Additionally, as described in previous research, patients were allowed to receive a higher quantity (MME) of opioids per prescription during the initial lockdown to help reduce the number of physical interactions needed with prescribers, to help prevent the spread of COVID-19.^2^ Our study did not adjust for this relationship of patients receiving a larger quantity of medication to limit in-person contact. Adjustments for other potential confounders were not applied. Another limiting factor to our research is that we are not able to determine whether a patient consumed the medication prescribed, because opioids are typically prescribed on an as-needed basis. Unfortunately, due to the nature of emergency response data for overdose incidents, it is not possible to link a specific opioid prescription directly to an opioid overdose event with certainty. This relates to our inability to account for opioid use that occurs outside of prescription fills, such as illicit, recreational, and nonmedical use.

## Conclusions

This study observed changes in the prescribing patterns that stemmed from the initial COVID-19 lockdown in March 2020 among both new and continuing prescription opioid users. It was observed that new users experienced greater changes than continuing users in terms of the number of individuals and the characteristics of the prescriptions following the onset of the pandemic lockdown. Specifically, there were changes in the average daily dose and the drug formulation prescribed. Both user groups had significant increases in the number of individuals receiving at least one prescription from a pharmacist, with continuing users seeing a notable proportional increase of 378.61%. Finally, there were no statistically significant changes observed across the prescription characteristics for individuals who had an opioid toxicity event. Although public health measures and lockdown restrictions were brought in with the best of intentions, there were likely many unintended consequences. The findings from this study will enhance our understanding of the potential associations of the COVID-19 response for non-COVID-19 health system resourcing needs.

## Supplementary Material

Supplemental Material
