# Supplementary material for: The Association between COVID-19 and Changes in Opioid Prescribing Patterns and Opioid-Related Overdoses: A Retrospective Cohort Study
Source: Can J Pain. 2023 Apr 6;7(1):2176297. doi: 10.1080/24740527.2023.2176297 (PMC10081074; doi:10.1080/24740527.2023.2176297)
Supplement: Supplemental Material [file UCJP_A_2176297_SM3301.docx]

Table 1. Full list of physician sub-specialties in the data provided by ICES. General Practitioner corresponds to GP/FP. Emergency Physician corresponds to Emergency Medicine and F.P./Emergency Medicine. Critical Care Physician corresponds to Critical Care. Other Physician corresponds to the remaining sub-specialties listed.

| ADOLESCENT MEDICINE |
| --- |
| ANATOMICAL PATHOLOGY |
| ANESTHESIOLOGY |
| CARDIAC SURGERY |
| CARDIOLOGY |
| CHILD & ADOLESCENT PSYCHIATRY |
| CLINICAL IMMUNOLOGY |
| CLINICAL PHARMACOLOGY |
| COLORECTAL SURGERY |
| COMMUNITY MED./PUBLIC HEALTH |
| CRITICAL CARE |
| DERMATOLOGY |
| DEVELOPMENTAL PEDIATRICS |
| DIAGNOSTIC RADIOLOGY |
| EMERGENCY MEDICINE |
| ENDOCRINOLOGY |
| F.P./EMERGENCY MEDICINE |
| FORENSIC PATHOLOGY |
| FORENSIC PSYCHIATRY |
| GASTROENTEROLOGY |
| GENERAL PATHOLOGY |
| GENERAL SURGERY |
| GERIATRIC MEDICINE |
| GERIATRIC PSYCHIATRY |
| GP/FP |
| GYNECOLOGIC ONCOLOGY |
| HEMATOLOGICAL PATHOLOGY |
| HEMATOLOGY |
| INFECTIOUS DISEASES |
| INTERNAL MEDICINE |
| LAB MEDICINE |
| MATERNAL FETAL MEDICINE |
| MEDICAL BIOCHEMISTRY |
| MEDICAL GENETICS |
| MEDICAL MICROBIOLOGY |
| MEDICAL ONCOLOGY |
| NEONATAL/PERINATAL MEDICINE |
| NEPHROLOGY |
| NEUROLOGY |
| NEUROPATHOLOGY |
| NEUROSURGERY |
| NUCLEAR MEDICINE |
| OBSTETRICS AND GYNECOLOGY |
| OCCUPATIONAL MEDICINE |
| OPHTHALMOLOGY |
| ORTHOPEDIC SURGERY |
| OTOLARYNGOLOGY |
| PAIN MEDICINE |
| PALLIATIVE MEDICINE |
| PEDIATRIC CARDIOLOGY |
| PEDIATRIC CLINICAL IMMUNOLOGY |
| PEDIATRIC CRITICAL CARE |
| PEDIATRIC EMERGENCY MEDICINE |
| PEDIATRIC ENDOCRINOLOGY |
| PEDIATRIC GASTROENTEROLOGY |
| PEDIATRIC HEMATOLOGY |
| PEDIATRIC INFECTIOUS DISEASES |
| PEDIATRIC NEPHROLOGY |
| PEDIATRIC NEUROLOGY |
| PEDIATRIC RADIOLOGY |
| PEDIATRIC RESPIROLOGY |
| PEDIATRIC RHEUMATOLOGY |
| PEDIATRIC SURGERY |
| PEDIATRICS |
| PHYSICAL MEDICINE AND REHAB |
| PLASTIC SURGERY |
| PSYCHIATRY |
| RADIATION ONCOLOGY |
| REPRODUCTIVE ENDOCRINOLOGY |
| RESPIROLOGY |
| RHEUMATOLOGY |
| SURGICAL ONCOLOGY |
| THORACIC SURGERY |
| UROLOGY |
| VASCULAR SURGERY |
